# Perceptions of plagiarism among undergraduate medical students in Rawalpindi, Pakistan

**DOI:** 10.12669/pjms.35.2.33

**Published:** 2019

**Authors:** Arslaan Javaeed, Abdus Salam Khan, Shafqat Husnain Khan, Sanniya Khan Ghauri

**Affiliations:** 1 *Dr. Arslaan Javaeed, MBBS, M.Phil, Department of Pathology, Poonch Medical College, Rawalakot, Azad Kashmir, Paksitan*; 2 *Dr. Abdus Salam Khan, MD, FACP, Department of Emergency Medicine, Shifa International Hospital, Islamabad, Pakistan*; 3 *Dr. Shafqat Husnain Khan, MBBS, M.Phil, Department of Pathology, Poonch Medical College, Rawalakot, Azad Kashmir, Paksitan*; 4 *Dr. Sanniya Khan Ghauri, MBBS, MRCEM, Department of Emergency Medicine, Shifa International Hospital, Islamabad, Pakistan*

**Keywords:** Consequences, Medical essays, Medical students, Plagiarism, Solution, Types

## Abstract

**Objectives:**

With the rise in the number of published papers in the biomedical field, plagiarism has become a major ethical concern as it has a direct effect on the quality of these papers. The objective of this research was to determine the perceptions of medical students towards plagiarism, the reasons students engage in plagiarism, the types of plagiarism, the consequences of plagiarism, and solutions to the problem of plagiarism.

**Methods:**

This is a cross-sectional study conducted in two medical colleges in Rawalpindi, Pakistan from June to September, 2018, using self-administered structured questionnaires.

**Results:**

Of the 1100 participants, up to 86.91% (n=956) were not aware of the existence of plagiarism, but the majority, i.e. 71.18% (n=783) have plagiarised the work of others before. Copying from colleagues or senior students is the most common type of plagiarism that medical students engage in owing to the ease with which fellow students’ work can be shared and copied. However, a lack of institutional awareness of the extent to which plagiarism exists, poor vigilance in detecting it, and the absence of clear policies to deal with plagiarism are mostly to blame.

**Conclusion:**

Plagiarism is common among medical students in developing countries, and it is necessary to create awareness about the consequences of engaging in this unethical practice both in the academic field and in the larger medical research society, in order to reduce its prevalence.

## INTRODUCTION

Plagiarism is an act of deliberately using another person’s work without giving acknowledgment to the person and to pass it off as your own without acknowledging the true source. Over the last ten years, there has been a rise in the number of identified plagiarised manuscripts that originate mainly from developed countries. This should not be taken to mean that plagiarism is absent in developing countries. Plagiarism in developing countries has not been researched, and therefore most medical students are not aware of plagiarism and its negative effect within the medical profession. Technological advances in developed countries have led to the development and use of plagiarism-detecting software which has consequently led to higher rates of reported plagiarism. The lack of technological advances in developing countries and consequently a lack of plagiarism-detecting software is responsible for the low rates of reported cases of plagiarism. This does not change the fact that professional attitudes and behaviours are critical in medical education.^1^ Our objective was to determine the perceptions of medical students towards plagiarism and the reasons students engage in plagiarism,

## METHODS

This study was carried out at two medical colleges in Rawalpindi, Pakistan from June to September, 2018. Undergraduate medical students from all academic years were randomly picked to take part in the study after obtaining their informed consent. The data was collected using a validated self-administered questionnaire. 2 The information was cleaned, coded and analyzed using SPSS Version 23.0 (IBM Corp., Armonk, New York, USA) into different measures of central tendencies. Chi-Square test and P values were also used to compare the association between variations with statistical significance. Ethical approval was secured from the ethical review committee of Poonch Medical College.

## RESULTS

Out of the thirteen hundred questionnaires, eleven hundred questionnaires were returned, recording a response rate of 84.61%. This study showed that 956 students (86.91) did not know about plagiarism, only 159 (14.45%) students were aware of the legal consequence of this practice, while 389 (35.36%) students considered it unethical, despite 783 (71.18) students admitting having engaged in plagiarism, as shown in Table-I.

**Table-I:** Knowledge and prevalence of plagiarism among undergraduate medical students (N=1100).

	Students	

	N	%
***Do you know what plagiarism is?***	
Yes	144	13.09
No	956	86.91
***Are you aware of the legal consequences of plagiarism?***	
Yes	159	14.45
No	941	85.54
***Do you consider plagiarism to be unprofessional or unethical?***		
Yes	389	35.36
No	711	64.63
***Do you practice plagiarism?***		

	***N***	***%***

Practiced	783	71.18
Not Practiced	317	28.81

The prevalence of plagiarism by gender showed that 401(51.21%) male students practiced plagiarism compared to 382(48.78%) female students. Table-II details the types of and reasons for practicing plagiarism.

**Table-II:** Types of and reasons for practicing plagiarism among undergraduate medical students (N=1100).

	Students	

	N	%
Type of plagiarism practiced*		
Copy & Paste of existing published material	217	27.71
Copying directly from peers	566	72.29
Reasons for plagiarism		
Ease of plagiarizing/laziness	435	39.54
Pressure to meet deadlines	428	38.90
Confusion	143	13.00
Cultural reasons	94	8.54

* Type of plagiarism practiced (those who admitted having practiced, n = 783).

The study, as depicted in Table-III, shows that medical colleges not only lack the resources to detect plagiarism, but also don’t have a punitive rules in place for this practice.

**Table-III:** Detection and response towards plagiarism by the medical college (N= 1100).

	Students	

	N	%
***Successful detection of plagiarism***		
Yes	153	13.91
No	947	86.09
***Response to plagiarism***		
Report to higher authorities	15	1.36
Lower grades	129	11.72
Warning	391	35.54
Ignore	565	51.36

The study revealed that very few medical students and their tutors have information about automated plagiarism detection software and their implementation. Only 12 (1.09%) medical students in the two colleges in Rawalpindi, Pakistan have heard of Turnitin^®^ (iParadigms LLC, Oakland, California, USA). Five (0.45%) of the students, on the other hand, have heard of iThenticate^®^ (iParadigms LLC) while 1095 (99.55%) have never heard about it .

## DISCUSSION

Universities and other institutions of higher learning provide a platform to generate new ideas, formulas, theories and standards through fieldwork, experiments and other research methods. These institutions are also developed to enable the production of highly competent and skilled individuals with exceptional levels of ethics, honesty, and professionalism.^3^ Since the prevalence of plagiarism cannot be measured directly with any accuracy, the study of students’ attitudes is investigated instead to give an insight into plagiarism. Ajzen’s theory argues that humans are rational and that norms are subjective. Attitudes and behaviour control are necessary for the performance of behaviour.^4^,^5^

Higher numbers of plagiarized papers are being detected and retracted from journals with the advancement in software to detect plagiarism. The increased ability to detect this unethical practice has helped to identify plagiarised manuscripts in journals.^6^,^7^ Despite this step, however, identifying plagiarized material does not reverse the damage that is associated with plagiarism. Although developing countries have not registered high prevalence of plagiarism, countries like Pakistan might actually have higher levels of plagiarism due to the lack of knowledge and information concerning the practice among medical students and their faculties, as evidenced by this study. However, intentional plagiarism cannot be ignored entirely as some students may have a sufficient understanding of ethical writing practices.

### Types of plagiarism practiced by medical students: ‘

Copy and paste’ is the most practiced type of plagiarism. Here medical students copy words, thoughts, and ideas from published and online sources without consent and/or acknowledgment of the original authors. For example, data from the study carried out in Pakistan shows that 217 out of 1100 students have copied and pasted from the internet without giving attributes to the owners of the research through appropriate scientific citation.^8^

Copying from peers is another closely-related but different form of this unethical practice where students copy from their classmates’ assignments. This is the most common type of plagiarism in institutions of higher learning as relevant work is rarely found on online databases or has been phrased differently from the tutor’s requirements. Another reason for such practice is the repetition of assignments by tutors over the years, thus prompting medical students to copy assignments from their senior peers who have previously carried out the same assignments.^9^

Closely related to plagiarism is fabricating data to achieve a certain advantage, such as justification for missing classes or fake submission of assignments. Medical students may forge their professors’ signatures; submit fake health certificates and fake results among other research. This type of behaviour is prevalent with medical students wishing to avoid repercussions for their actions.^10^

### Reasons medical students engage in plagiarism

When medical colleges are unable to detect plagiarism in students’ manuscripts, the students usually not informed on the consequences of plagiarism such as poor marks or even dismissal from the learning establishment in an academic environment, to more legal consequences such as fines or even imprisonment. Thus, they engage in unethical academic behaviour through ignorance.^11^,^12^ In this study, out of 1100 participants and the 783 who confirmed they had engaged in plagiarism, only 153 of them reported that it had been detected.

Top of the list of the reasons for such unethical behaviour is the ease of plagiarizing papers, where copying from their peers’ simply saves time. Plagiarism does not require any effort, and therefore most students opt for it instead of writing original material.^13^ Pressure to meet deadlines may lead to student’s inability to complete their assignments on time and therefore they end up copying work from their peers, journals or online sources. Poor time management will push students to seek the easiest way to finish their work before deadlines. Due to fear of failing, medical students may resort to means that will ensure they pass their exams by copying from the students they think are better achievers or copying directly from the internet. Inability to understand their assignments may also lead medical students to copy work from their peers or online sources.

Cultural reasons, such as having to study in a different language to their native one may push medical students into copying assignments as those who are not native speakers of a particular language may not be familiar and/or competent in the required language. Further to this, students come from all regions in Pakistan to the colleges in Rawalpindi. The lack of prior knowledge on plagiarism is the primary reason why the prevalence of plagiarism in Pakistan is at such a high rate. Not every student comes from an environment that instils social norms, such as honesty, and therefore plagiarising may not seem unethical and bad practice to them.^14^ Confusion arising from exposure to different methodologies of tackling academic papers may push students into copying from peers or online sources.^15^

### Consequences of plagiarism among medical students

A high number of research articles that are being retracted from journals every day are because of image manipulation, faked data, fake peer reviews, self-plagiarism and authorships that are disputed. This dishonesty is not the sole domain of researchers and medical students, but also includes post-doctorate and doctorate scholars from all over the world who enjoy international prestige and fame.^16^ Using people’s work without due attribution often results in major setbacks in a person’s career.

Professional attitudes and behaviour are critical in the study of medicine. Practices that are unprofessional among doctors means that patient safety can be put at risk, working relationships can be compromised, while unethical behaviour can lead to disruption of normal medical activities. Medical students who engage in unprofessional learning activities such as plagiarism may be removed from academic programmes, stripped of their qualifications, or become fake doctors. Furthermore, legal action can be taken against these parties as they may be sued for deceit and malpractice.^17^

### Solutions suggested to reduce the prevalence of plagiarism in medical colleges

Medical ethics training should focus on discouraging plagiarism in medical writing. The need for integrity and honesty in the profession should be taught in medical colleges. Developed countries have incorporated this training, and medical ethics is currently among those topics being studied.^18^,^19^ Developing countries are also trying to create awareness about the importance of the medical ethics. For example, the Pakistan Medical and Dental Council encourages that a physician should always ensure they maintain the highest standards of professional conduct, conform to principles of justice and honesty, and recommends that all dental and medical institutions and colleges include medical ethics in their curricula and this is extended to practicing health workers.^20^ Academic integrity can also be instilled through effective student-teacher relationships as shown by a study done at the University of Punjab.^21^,^22^ When teachers make it clear that avoiding plagiarism in medical essays enhances honesty in practice, students are likely to emulate this behaviour. By ensuring that teachers reprimand students when they are caught cheating, particularly through plagiarism, students will avoid engaging in plagiarism to maintain a good relationship with their teachers.

Outlining the consequences of plagiarism will ensure medical students will be more likely to avoid such a practice. Clearly describing the types, definitions, incidences and consequences of plagiarism, ways of preventing plagiarism and the penalties associated with plagiarism, through seminars, lectures and workshops will help in reducing the prevalence of plagiarism. Setting examples to medical students by actually carrying out punishments on those found to have plagiarised the work of others will raise awareness and reduce its prevalence. By creating an ethical environment, this will push the students to embrace it and emulate it.^23^ Places of learning should also invest in software that detect plagiarism in papers submitted by medical students. Such software includes; iThenticate® (iParadigms LLC) and Turnitin® (iParadigms LLC, Oakland, California, USA).^24^,^25^

## CONCLUSION

The study shows the presence of a high level of plagiarism among medical students due to a lack of awareness of the unethical practice and its consequences. There are also lower rates of detection of plagiarism in institutions of higher learning. It is clear that providing training on integrity by avoiding plagiarism in written works and explaining the consequences related to plagiarism will reduce its prevalence among medical students. Enabling students to understand the ethical standards required in medical writing will also curb the practice of plagiarism.

### Author`s Contribution

**AJ, ASK** conceived, designed and did statistical analysis & editing of manuscript.

**SKG** did data collection and manuscript writing.

**AJ** did review and final approval of manuscript.
